# Accuracy as a Condition of Scientific Progress

**Published:** 1886-12

**Authors:** J. Smith Dodge

**Affiliations:** New York.


ARTICLE II.

### ACCURACY AS A CONDITION OF SCIENTIFIC PROGRESS.

BY J. SMITH DODGE, JR., M. D., D. D. S., NEW YORK.

[Read before the Dental Society of the State of New York, May, 1879.]

### A RETROSPECT.

The number of things which a practicing dentist ought to know threatens to become overwhelming. Those who can remember the novelties introduced within the last twenty-five years will be equally astonished, on making the survey, that such a revolution could occur in such a time, and that any art could endure such a revolution and yet remain essentially the same. It is now about twenty-five years since the profession began to appreciate the cohesiveness of pure gold, and to use this quality in filling teeth. One of the first results was a demand for more perfect dryness than any previous means afforded, and the imperative demand produced that master-piece of invention, the rubber dam. But the rubber dam does not always choose to stay where it is put, and further invention easily produced the numberless brood of clamps and their adjuncts, until the problem of dryness was fully solved, and one of the greatest difficulties of careful filling absolutely abolished. From the first suggestion of cohesive filling, the more daring minds had conceived of operations never before imagined; and with each added facility the ideal grew more and more exacting. Of course, new tools were requisite to perform these new operations, and the burring-engine inevitably followed. But the engine, invented to do what had been previously conceived, presently enlarged, without limit, the conception of what might be done. It now became possible to do to a tooth, within practicable limits of time and labor, anything of a mechanical nature which the operator might desire; and as the engine put the form and substance of the tooth wholly within the dentist's power, so the whole range of malleting inventions—from blows that would nail a box to the vibrations of the electric mallet—made it possible to give any conceivable bulk and shape to fillings.

Now, all this is a bare outline of only a single course among others along which dentistry has been developing. If one proceeds to fill in this outline with the infinite varieties of filling materials, hand instruments, office furniture and other appliances which group themselves naturally about these salient features, he will begin to grow bewildered. But when all this is done there remain at least two coordinate lines of progress not yet suggested and equally prolific. Quite as full a statement might be made of the evolution of mechanical dentistry which touches at so many points various industrial processes of general application. And again, on the other side of operative dentistry, spreads the vast field of anatomical, physiological and histological research, industriously cultivated through all these years, and fruitful of results which may, indeed, seem remote from our daily work, but which some new pathological condition may, at any moment, thrust imperatively before one's face as the one thing he needs now to know. But even when these three strands are twisted into a single conception, the whole is not stated. For this process of advance rolls forward with unceasing increase. Every capital discovery comes to us pregnant with an unimaginable brood of auxiliary novelties, and so far are our present attainments from completeness that each of them seems an open door into the future, and there is probably no one thing of which all dentists are so sure as that the progress of the dental art has only begun.

### THE OUTLOOK.

If we sweep, then, with a comprehensive view, the entire field—past, present, and immediately to come—it will appear no exaggeration to repeat that "the number of things which a practising dentist ought to know threatens to become overwhelming." And the difficulty is the graver, because this advance goes on by a certain organic force of its own, developing new activity at most unexpected points, and remaining stagnant for long periods just where we most desire improvement.

No man can say or guess what theory of his will be exploded next week, nor what favorite method of practice may have to be dropped, and a new one learned instead. It is all we can do to hold our ground and yet, at any moment, the bugle may sound an advance. For a conscientious man all this is no small perplexity, and he may well ask himself and his neighbors what must be done.

Fortunately, we are not the first among men to meet the same trouble, and the way of escape, while it is not easy, is well known. The growing intelligence of mankind, since the dawn of the twelfth century, and especially for the last four hundred years, has been beset by a swelling flood of individual facts in all departments of knowledge, and has had, in respect to the entire field of human observation and action, just that difficulty of retaining and using its acquisitions which now besets the narrower area of the dental profession. And mankind long ago found the solution of the problem which is called Science. The urgent need of dentistry at this day is, that it become, not so much a separate science, for it is too composite for that, but through and through, and in all its details, scientific. For science is like the fairy tent in the Eastern tale, which could be held at need in the palm of one's hand, and yet expanded in another moment to shelter an army. Science may, this morning, be focalized upon the slender margin of a proximal filling, and this afternoon formulate the universe.

### WHAT IS SCIENCE?

Now, what is science? Many persons, who ought to know better, imagine that a man of science is one who knows more or less facts generally unknown, and can state strange theories about them; so that remoteness from the observations and conclusions of average life, becomes the central idea of science. How absurd this idea is need not be argued before this assembly; and yet even those who are doing honest scientific work do not always know what constitutes a valid claim to the honor of that name. Etymologically, science means, and has meant, these five and twenty centuries, *knowledge*. But, in the language of the nineteenth century, *science is the knowledge of things in their relations to each other*. And this recognition of the supreme importance of *relation* in the knowledge of facts is the distinctive mark of all science. The mechanic, the miner, the savage hunter, can teach the physicist, the geologist, and the naturalist, separate facts which the learned never knew. But to the empirical minds of the former each fact stands by itself—curious or useful, but alone; while the man of science immediately fits, or strives to fit, each separate fact into some gap where other facts had not seemed completely adjusted to each other; and the result is often like adding the keystone to the arch—it is only one stone more, but it makes efficient all the rest. Precisely the same consideration, turned the other way, corrects the pernicious fancy that the chief business of science is to spin theories, and mostly from a very slender staple. So untrue is this that the moment any man's theory goes a hair's breadth beyond known facts, science refuses to be responsible for the conception, and it becomes the individual hypothesis of the inventor, to stand or fall on his responsibility alone. What is actually done, when the wonder-working hand of science ennobles the meanest thing, and brings cosmos out of chaos, is to demonstrate the ascertained relations of ascertained facts, constituting an order which existed equally before it was suspected, and is wholly independent of human invention. There is no more perfect statement of the true meaning of scientific disccovery than Kepler's, who, having at last verified the three laws of planetary motion, exclaimed, "O, God, I think thy thoughts after Thee!" And this idea, which is so sublime in its larger applications, abates nothing of its severity when it is applied to the smallest and most familiar things. In the contour of a bicuspid, as in the movements of the heavens, that, and that alone, is scientific which expresses the actual relation of actual facts.

It is by thus *sorting* our facts that science removes the vast difficulty of remembering or recalling the numberless integers of human knowledge. That fundamental law of memory, association of ideas, enables the mind to pass from point to point along and across the endless web, and having what is before the eyes, to recall whatever of the kind has been previously seen. By this means alone, the human mind is capable of the knowledge it already possesses, and looks forward, without dismay, to the sure increase of the future. And it must be by this means alone that dentistry will keep at its free disposal all the past has yielded, and be glad to give hospitable welcome to all that is yet to come.

ACCURACY.

From this point one may diverge into many profitable trains of thought. It is the object of this paper to follow only one of them, by insisting upon that habit which is at the core of all scientific advance, and which, the writer makes bold to say, is to-day the one most striking deficient in the mental processes of dentists—accuracy. In this connection, accuracy means simply keeping close to the facts, whether they be facts of being or facts of relation; and all that has been said shows the supreme importance of that posture. The scientific knowledge of the universe is like a Waltham watch, a complicated fabric of many parts, each constructed by a different man in a different room. The parts being done, the watch is done, on the one condition that each part is exactly right. If this is so, all will go smoothly together and keep perfect time; but if any one workman has made his piece a trace larger or smaller than the gauge, the piece will not fit and the watch will not run. So if any man reports, under the name of science, a fact a hair's breadth wider or narrower than the gauge of exact truth, it will frustrate some other man's observation and throw the whole group to which it belongs into confusion. But it is the characteristic of truth, exact truth, that it "fits all around." So that a fact accurately reported a thousand years ago will tally exactly with its correlative fact discovered yesterday.

With these preliminary generalties let us now consider more in detail several ways in which our art demands more scientific accuracy than it has habitually found in its votaries. But here let me disclaim the attitude of one who passes judgment on the fault of others. The difficulty of maintaining always that accuracy which is the vital breath of science is so great, that no man need hesitate to own he has many times failed in the endeavor. Certainly the writer owns it freely, for the faults which are to be criticised are best known to him from the efforts he has made—not always with success—to avoid them.

### KNOW THYSELF.

The first demand for accuracy which the dentist is bound to recognize, relates to his own estimate of himself. When the Rev. Jasper stoutly asserts, "The sun do move," and preaches sermons to demonstrate that it is on the east side of the barn when it comes up and on the west side when it goes down, that dusky divine is beyond question perfectly honest, and his chain of evidence seems to him complete and unanswerable. His mistake simply is that he supposes himself fit to judge of things for which, in fact, he has no capacity. He neither knows the amply demonstrated facts which prove the rotation of the earth, nor has he the mental training necessary to understand the cogency of their meaning if the facts were laid before him. The difference is that most Americans are intelligent enough to know their own position in such matters, and do not presume to question the consenting testimony of all those who are competent to judge. To this modest and correct self-estimate Mr. Jasper is the exception. Now, among dentists the Jaspers are uncommonly many. We are generally a self-satisfied and willful class, and each of us is apt to think he has seen all the human mouth has to show and is a little better qualified to judge of it than anybody else. While the fact is, as every educated man among us will confess, there are but few (besides himself) who can impartially observe, carefully generalize, verify by satisfactory experiment, and so add to the accurately ascertained science of our art. Perhaps this misconception was at first unavoidable. When the appeal was made to dentists to study their facts and lay a scientific basis for dentistry, it was natural the apostles of science should declare no man was fit to practice who was not a scientific man. And if this was the word, then multitudes of dentists who had honestly plodded along before and done much good, mixed with some unmeant harm to mankind, must decide whether to own themselves unfit for the work which fed them or to become "scientific." Of course they decided upon the latter. It was rather interesting and not too laborious to read a few books and remember the long words, to look over some plates and peep into a microscope now and then, and presently to select some one of those thousand fancies which swarm in all dentists' heads and vent it decked with learned terms as a guarantee of scientific standing. The hands, indeed, seemed the hands of science, but the voice was the voice of Jasper.

The root of the whole mischief lies in one ambiguity. Dentistry is an art, and is striving to become a science. And every dentist is bound to sympathize with this endeavor, to keep himself informed of all progress that may be truly made in it, and, *if he be competent*, to help it on. But it does not at all follow that a man's practice is worthless and he a charlatan because he cannot give scientific account of what he does. There is many a man whom I would gladly have fill a proximal cavity in my molar, but for whose opinion upon the electro-chemical theory of decay I do not care a fig. And it would be infinitely more dignified and honest for such a man to listen quietly, doing what he has found good until something better is shown him, than to rush into speech or print with his crude and worthless opinions. They encumber the field and bring the discussion into contempt. But the manipulations of the dental art, honestly performed according to the operator's experience or that of his teacher, while they may be called old fashioned, will do service to his patient and win honor for himself, though he should declare himself continually a learner and not a teacher of science.

LEARNING AND TEACHING.

But supposing this selection to be somewhat fairly made, there remains much need to urge accuracy upon those who are qualified to push the car of *Science*. And for these the subject may be divided into two branches accuracy in learning and accuracy in teaching; the former covering the process by which the investigator ascertains scientific truth—the mental endosmosis, and the latter that by which he conveys to other minds the knowledge he has acquired—the mental exosmosis.

The process of acquiring scientific knowledge at first hand, consists of three parts, the observation of facts in nature, the deduction of a generalization from a series of related facts, and the verifying of this generalization by suitably contrived experiments. The student of dental science has peculiar advantages in pursuing this process, because the successive steps are, in his field of observation, easily distinguished. Let us take each step in order.

*Observation.* It is one of the popular fallacies that nothing is easier than to see what is before one's eyes. But the fact is there is nothing more difficult. Hardly a suit is tried at law in which eye-witnesses of equal credibility do not contradict each other concerning simple facts. There is probably not a dentist in this assembly who could at this moment describe the conformation, tooth by tooth, of the full human denture, without making blunders which a dozen others would immediately detect. And yet each of the dozen in his turn would make other blunders equally great. It will be a wholesome lesson to any who may doubt this, to try the experiment of studying his watch-case or his pen-knife five minutes, and then attempting to write an accurate description. Now, it is just this *accurate* account which science needs. Give this, and it will fit any other observation or any continent. But the untrained mind imposes upon the mental impression which an object makes, such distortions as its prevailing habit of thought or its present mood or passion happens to dictate, and the untrue result passes into memory as an actual presentation of the object. But this is not scientific observation. On the contrary, it is necessary that the scientific observer be on his guard against these delusions; and even with long practice he will hardly acquire such a habit of seeing simply what is before his eyes as will relieve him of the duty of cross-examining his perceptions while the facts are still in sight.

It can hardly be necessary to insist much upon the statement that the annals of dental observation are vitiated most broadly by this unpardonable fault. How else are we to take those frequent marvels which are gravely related as common in this or that gentleman's practice, but which nobody else ever sees? It is impossible to imagine that dentists lie, but it is certain they squint terribly. Let me again repeat that the difficulty is enormous of eliminating from our simplest observation that which others have reported, that which we hoped to find, that which our dyspepsia or our weariness or our vanity suggests, and seeing merely that which we see. Let no man feel offended at the suggestion that he needs this purging of the eyes, or ashamed to confess that he finds it extremely hard to achieve. Accurate observation of separate facts is the corner-stone of science, and only master masons can lay it.

*Generalization.* The mind is constructed with an irrepressible tendency to generalize. Two facts can hardly be observed in succession without a perception of some likeness or unlikeness, and as the series extends the comparison grows into general ideas. In fact the mind does this work extensively and with much correctness without our conscious effort. Men who never deliberately deduced a principle from a series of facts, nevertheless have general ideas by which they guide their conduct and to a degree predict the future. Probably a close scrutiny would show that all minds necessarily depend much upon the results of such unconscious cerebration. But while the process is largely unconscious, it is still governed by those habits of mental action which we have full power to train and cultivate. He who takes care to think closely when he is purposely thinking, will find his mind has done its spontaneous work in an equally accurate manner. While he who permits his desire or his sloth to vitiate his deliberate thought, will find his mind slovenly and untrusty when it works of itself.

Now, by this process of generalizing, the mind, having observed a certain thing in a certain relation again and again, relieves itself of the burden of remembering each instance separately by formulating the whole—A implies B. A swelling under the gum and certain reports of pain make the dentist as sure of an alveolar abscess as if he had extracted the tooth and seen the sac. The first imply the second. And this process of distilling the active principle from a series of facts, is the precise and constant function of science. It is the knowledge of things in their relations. Here, therefore, is the utmost need of accuracy. But here, also, is the utmost danger. The process is so fascinating that it is especially hard to keep the mind in suspense when a few data tempt it to assume rest in a general statement. All sciences, from chemistry to theology, are loaded down with these hasty conclusions of former times. And a great part of the business of scientific thinkers at this day is to test anew accepted principles and eliminate venerable errors.

### NIMBLE-MINDED DENTISTS.

Of course a set of men so nimble-minded as dentists, have not been backward in this charming pursuit. No part of our history, and least of all the present, has lacked abundant instances of inaccurate generalization. Sometimes the error has lain in the faulty observation of facts, so that one may say the theory would be correct if the facts were only as they have been understood. But this goes back to the first step in the process of scientific investigation ; and of that enough has been said. The inaccuracy peculiar to the process of generalizing is the deduction of general statements from too few observations. If some dentist to the Bourbon family should declare the protruding lower jaw a normal conformation, because many of his best patients had it, he would be settling the standard of fourteen hundred million people from a dozen observations ; but he would be little more hasty or inaccurate than many dental theorizers have been. Three faulty deductions, current in three epochs of dental practice will make clear the nature of this error and the need of making sure the basis of our general statements.

Long ago this maxim was somewhat current : "A tooth which has ached cannot be saved." We smile at that now, and probably it was never held in its full meaning. But it was honestly meant as the summary of many fruitless attempts to manage exposed and inflamed pulps, mostly ending in disaster. The disappointed dentist might naturally conclude to ignore the rare exceptions and extract aching teeth. The error obviously lay in assuming that the limited resources of that day covered the whole possible ground ; and the vice of this assumption readily appears if we consider on the one hand the very narrow limits of dental manipulation and dental medicine at the time, and on the other the large results which have gradually come from disbelieving this rule and trying for better ways.

Far more recently another maxim has had many advocates : "Any tooth that is worth filling can be saved with gold." If the New Departure has done nothing else, I fancy it has killed that idea. But most dentists who have had experience enough to know the value of a tooth, have been gradually coming of late years to care less what a tooth is filled with and more for the result. To my mind it seems clear that these worshipers of gold really meant, if they had said their exact meaning, that no tooth is worth filling which gold will not save. Their error, therefore, lay in excluding from their view all teeth but a single group, and from the latter drawing a principle for universal application. Many a well eaten dinner to-day will give, against their narrowness, the testimony of despised but useful grinders which gold could not have saved.

THE NEW DEPARTURE.

My third instance of hasty generalization is as novel and noisy as a new-born babe. It is the first article in the creed of the New Departure : " In proportion as teeth need saving, gold is the worst material." Since this startling law was announced I fancy many hundred dentists, as they made the customary examination of mouths long under their charge, have wondered on what evidence the statement rests, and pondered over those mystic vials in Philadelphia. I remember a central upper incisor which split from side to side, throwing off the entire face from the groove on the cutting edge to a point far under the gum, and laying open the empty pulp-chamber. The mouth was well filled with good teeth, and it seemed as if the need of saving that tooth was of the largest proportion. It was nothing wonderful to replace the lost half with gold, nor was I surprised to find it lately in perfect condition, after six years' service ; but considering the great need this tooth had of filling, I was puzzled to understand why gold was the worst material that could have been used. And we should stay here a week if every dentist present gave the many cases of equal plainness which he can recall. Confronted with these every-day facts, the Departing brethren would doubtless say they did not mean these, and on cross-examination it might probably come out that their minds were chiefly fixed on the *cervical* borders of proximal fillings in bicuspids and molars. To which injured and hindered Science would reproachfully answer, "Then why did you not say so?" It can hardly be doubted that the daily experience of the profession will soon make plain that this latest novelty in generalization is the fruit of minds which have so much concentrated their gaze on certain facts, as to forget all others and make the part equal to the whole. It may seem that too much space has been given to this topic. But no space can be too great if it impresses upon our minds the exceeding difficulty and importance of accuracy in drawing general ideas from individual observations. And I will take the liberty of suggesting to our teachers that they should *incubate* long and patiently over their facts before they presume to announce what manner of fowl they have hatched. Meanwhile, nothing will be more useful or more scientific than a constant familiarity with certain qualifying words and phrases:—"Probably," "In my opinion," "So far as is known."

*Verification* by experiment, is the final step. And here the branch of operative dentistry has all the advantages of an applied science dealing with familiar things. When Columbus had inferred the rotundity of the earth, it took him many years of toil and delay before he could try the experiment of sailing westward. When Ericsson had deduced the idea of the screw propellor, it required governmental aid, which was not easily obtained, to test the invention on a satisfactory scale. But when the operative dentist has slowly worked out a new idea relating to his art, he may confidently expect within a week or two the chance of putting it to the test. Every trial of a new material or a new instrument, every new shaping of a tooth or a cavity, every new medicine laid on a pulp or injected into an abscess, is, in the best and scientific sense of the word, an experiment, provided only it be made not at hap-hazard, but with a definite theory to test, and an intelligent choice of cases. Add to these the experiments of the laboratory, which are so happily becoming frequent among us, and the observations of the microscope and the scalpel, and it cannot be said that dentists are neglecting this crowing portion of scientific research.

But it must be said that while much of this is done daily, and while dentistry is making much sure progress by these means, still we are beset by theorizers so ignorant of scientific methods that they suppose the whole process to be complete when they have looked at facts and spun from them a more or less careful theory. Then the noise begins, and the wonderful discoveries are announced which startle us to-day and amuse us to-morrow. Let us all remember that science holds to facts at both ends. Her work begins with observing them as nature presents them, and ends with submitting to their inexorable decision under experiment whatever she has inferred. So that there is not and there never can be a single theory or general statement of anything in the universe which is a part of true science, except those which have been fully verified by the test of experiment. All besides is opinion or conjecture, more or less useful and often the best we can attain for the present, but wanting something still to make it scientific truth. But he who has patiently gone through this process, has accurately observed, accurately generalized and accurately verified, has enlarged the boundaries of human knowledge, and has claim to the respect and honor of mankind, whether the object of his research be the axis-cylinder of nerves or the order of the heavens.

### KNOWING AND TELLING.

It remains to consider the converse process of making known to others that truth which has been gained in this laborious way. The two faculties do not necessarily go together. Because a man has learned, it does not follow that he can teach. On the contrary, the writer has known a maker of artificial dentures whose skill in all the details of constructing and adapting his work was simply marvelous, the admiration of all beholders. In his laboratory every tool was exactly right, and every process strictly methodical, while his results were always of the highest quality. And yet this man was wholly unable to describe his methods. In the attempt he would omit essential details, and at last break down in failure, saying, "I can show you, but I cannot tell." Now, the interests of science require that such a man should be silent. It is no impeachment of his one excellence that he has not a certain other excellence; but it is a grave fault if because he can *do* he therefore claims an undeserved privilege of *saying*. His words will be inaccurate, and his half-told truth will have the effect of a lie. But what a few do from a fault of natural capacity, many do from negligence. Part of a process is told with the unconscious assumption that the hearer will supply what is not told, whereas, the hearer has no hint that anything is left to be supplied. Cases are stated without mention of surrounding conditions, although these conditions are essential to a just conclusion. But there is no need to multiply applications. It is among the commonest experiences of those who look into new dental discoveries or methods, that when they see the *thing* the first glance shows some essential deficency in the description. "You didn't mention that," says the spectator. "Why, I thought anybody would understand that; of course," answers the would-be teacher. A notable instance of this is recorded in the published minutes of this body for a year not very remote. A gentleman read a paper describing his method of treating exposed pulps. He was understood to claim that by this method he saved almost a hundred per cent. of all cases which came to him. Such a claim, of course, called forth a brisk fire of cross-examination, which at least drew out the fact that the writer only attempted such cases as he judged would do well, and *of these* he saved the large percentage named. So that the gentleman's paper demonstrated, not the success of his method, but the shrewdness of his judgment. These and a hundred other instances present the common characteristic of confused mental conception as the cause of inaccurate teaching; and the remedy is to be found in mental discipline.

OUR VOCABULARY.

But there is a second cause which obscures literature, and which is less excusable. Some of our instructors who seem to know well enough what they want to say, fill their teachings with unusual or even unknown words. Having constructed a theory to express, they next construct a language to express it in ; forgetting, however, to furnish the hearer a dictionary of the new tongue. It is of course true that the necessities of *science* call for many new words, partly because new things are to be named, and partly because an accuracy of expression is needed, which is not easily attained with common words. But much of this is already done, and the results are at our service, so that it is a positive fault to distort the statement of important facts by the use of obscure and uncouth substitutes for accepted words. If the gentlemen who find themselves subject to this temptation would study the dictionary, they would probably be surprised to find how amply the English language is already provided for all occasions, and their hearers would certainly be delighted at finding themselves able to understand every word the teachers say.

Finally, to sum up this extended paper in few words, we dentists are confronted with a multitude of things already known and another multitude, which no man can number, of things pressing to discovery. The only possibility of having all these at our command lies in knowing them *in their relations*, which is science. And the fundamental requirement for obtaining such scientific posession is *accuracy*, —accuracy of eye and touch, accuracy of perception and memory, accuracy of comparison and induction, accuracy of test and accuracy of speech, in a word such accuracy of the whole observing man as may be worthy to set itself beside that infinite accuracy of fact which we call the Laws of Nature.—*Odontographic Journal.*

